# Macroporous 3D Chitosan Cryogels for Fastac 10EC Pesticide Adsorption and Antibacterial Applications

**DOI:** 10.3390/polym14153145

**Published:** 2022-08-02

**Authors:** Ionel Adrian Dinu, Luminita Ghimici, Irina Elena Raschip

**Affiliations:** “Petru Poni” Institute of Macromolecular Chemistry, Grigore Ghica Voda Alley 41A, 700487 Iasi, Romania; lghimici@icmpp.ro (L.G.); iecoj@icmpp.ro (I.E.R.)

**Keywords:** chitosan, cryogel, Fastac 10EC removal, adsorption, antibacterial properties

## Abstract

The pesticide pollution of surface water and wastewater has been recognized as a major worldwide concern due to their persistence in the aquatic environment and the potential adverse effects on human, flora, and fauna health. Apart from pesticides, bio-contamination with various bacterial populations leads to waterborne diseases. Hence, it becomes vital to remove the above-mentioned pollutants from water using a suitable process. Consequently, our study emphasized the potential benefits of a highly porous, chemically cross-linked 3D chitosan (CSGA) cryogel in the removal of pesticides and bacteria. The CSGA sponges were prepared using a facile and cost-effective approach that consisted of a three-step cryogenic process: (i) freezing at −18 °C, (ii) storage in a frozen state for a certain period, and (iii) thawing at room temperature. Batch adsorption experiments were performed under different environments, where the effects of several parameters, such as pH, contact time, and initial pollutant concentration were evaluated to identify the appropriate adsorption conditions for maximum pesticide removal. The CSGA-based cryogel sponges exhibited a theoretical maximum adsorption capacity of 160.82 mg g^−1^ for the Fastac 10EC pesticide and very good recyclability at room temperature. In addition, the antibacterial activities of these sponges were also investigated against various bacterial pathogens. The rates of killing *Escherichia coli*, *Listeria monocytogenes*, and *Staphylococcus aureus* were close to 82%, 100%, and 99%, respectively. These results demonstrated that CSGA cryogels could be efficiently used in water remediation and find applications in the removal of pesticides and disinfection.

## 1. Introduction

Pesticides have become essential for successful modern farming to protect crops and livestock, which are vital to our food supply and economy [1]. However, the extensive use of pesticides in agriculture has also resulted in serious consequences for health. These contaminants have a harmful impact on humans by causing adverse health effects, including cancer, infertility, malformations, and chromosomal mutations. Numerous studies showed that pesticides not only affect the target organisms but also the whole environment, including the atmosphere, soil, groundwater, and surface water [2,3]. Consequently, over the years, a variety of different technologies, including biological treatments with strains of fungi and bacteria, chemical remediation based on advanced oxidation processes (UV-H_2_O_2_ and UV-ozone, Fenton reaction, photocatalysis, and so on), and adsorption processes, were proposed and explored for the removal of pesticides from polluted water [4,5]. Unfortunately, all these processes have their associated drawbacks, including high operational costs, the use of harsh chemicals, and the generation of secondary waste. Therefore, there is an urgent need for a low-cost, environmentally friendly, and highly efficient approach to the decontamination of the effluents that result from agricultural activities. Thus, raw materials, such as activated carbon [4,6], rice bran [5], bagasse fly ash [5], clays [4,6], and zeolites [4,6], were reported as sorbents for pesticide removal. Increasing attention has also been focused on polysaccharide-based biosorbents and their composites due to their easy availability, bio-compatible nature, non-toxicity, and high efficiency. Considering these advantages, a plethora of modified polysaccharides based on chitosan (CS), dextran, cellulose, pullulan, starch, alginate, etc., were successfully used as flocculants or adsorbents for the removal of various contaminants, including clays [7], zirconium silicates [8], metal oxides [9,10], metal ions [11,12], dyes [13], oil [14], bacteria [15], and pesticides [16,17]. By comparing their adsorption properties, CS (*β*-1,4-linked polysaccharide) has received significantly increased attention among the options available for its ability to remove heavy metal ions and organic pollutants from wastewaters [18,19,20,21] due to the presence of –NH_2_ and –OH functional moieties on the glucosamine repeating units [18,19,20,21]. The application of CS-based sorbents for the removal of different classes of pesticides was also demonstrated [22,23,24,25,26]. Thus, methyl parathion [22] and permethrin [23] were successfully removed using silver-complexed CS microparticles, whereas clopyralid [24] was adsorbed on CS-clay nanocomposites. CS beads and crab shell powder were used as sorbents for the removal of organochlorine pesticides [25], while CS films allowed for the retention of an organophosphorus pesticide [26].

Conventionally, the abovementioned CS-based sorbents were synthesized in bulk gel form and then dried and crushed to get particles of the desired size. However, the particles obtained in this way usually have a lower mechanical strength (they cannot be reused in another adsorption cycle) and an irregular size, which make them unsuitable for proper column operation. Moreover, there is a high probability that the sorbent particles may remain suspended in the treated water, and thus, they act as a contaminant. In addition, the use of sorbents fabricated as compact films is limited by the poor diffusion of contaminants to the active sites. The disadvantages associated with the conventional approaches may be overcome when the CS-based sorbents were synthesized directly in the bead or monolith form and possess a tailored porous morphology. In our previous investigations, we used cryogelation to prepare CS-based porous composite hydrogels as monoliths and their remarkable potential as green sorbents for the removal of heavy metal ions from aqueous solutions and as low-cost catalysts for the degradation of dyes was demonstrated [12,27,28,29]. This straightforward method for the preparation of porous composites, along with their excellent stability and high efficiency during regeneration and reuse cycles [27,28,29], motivated us to extend the application range of CS-based materials to pesticide removal. To the best of our knowledge, there are no data reported so far regarding the use of CS-based cryogel for the adsorption of pesticides.

In this work, we report the physicochemical characterization of CS-based cryogel monoliths with an interconnected porous structure and their adsorption efficiency. To evaluate their potential as sorbents, we investigated the removal of the Fastac 10EC pesticide from an aqueous emulsion. Fastac 10EC is the commercial formulation of *α*-cypermethrin, which is a highly active broad-spectrum pyrethroid insecticide that essentially consists of two of the four cis isomers comprising cypermethrin. This pesticide is effective against target pests via contact and ingestion, and it is widely used on crops, in forestry, and in public and animal health. The effect of several experimental parameters, such as the amount of sorbent, initial pesticide concentration, contact time, and pH, on the adsorption efficiency was studied in detail. Furthermore, we were interested in evaluating the antimicrobial activity of CS-based cryogels against *Escherichia coli*, *Listeria monocytogenes*, and *Staphylococcus aureus* bacterial pathogens. The obtained experimental data were further modeled and optimized to provide useful information for designing effective and low-cost wastewater treatment plants.

## 2. Materials and Methods

### 2.1. Materials

**Figure 1:**
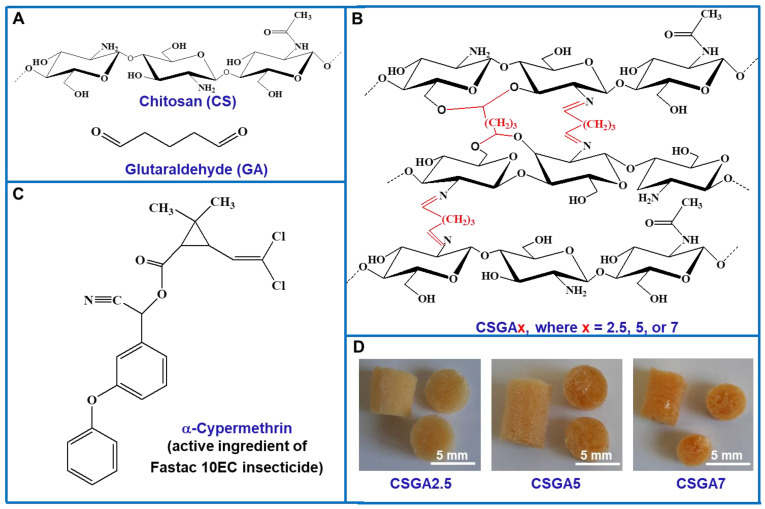
(**A**) Chemical structures of chitosan (CS) and glutaraldehyde (GA). (**B**) Schematic representation indicating the chemical structure of the cryogel sorbents (the cross-linking with GA is shown in red). (**C**) Chemical structure of *α*-cypermethrin, which is the active ingredient of Fastac 10EC pesticide. (**D**) Digital optical images of the cryogel sorbents as monoliths, which differed by cross-linking degree (*v*/*v*%).

All reagents were of analytical purity. CS, with a viscometric average molecular weight of 467 kDa and 15% acetylated units, and glutaraldehyde (GA), as an aqueous solution with a concentration of 25%, were purchased from Sigma-Aldrich Chemie GmbH, Darmstadt, Germany. Fastac 10EC (BASF Agro B.V., Arnhem, The Netherlands) is commercially available in vials of 2 mL solution (*α*-cypermethrin: 100 g L^−1^). Synthetic emulsions of Fastac 10EC were prepared in deionized Milli-Q water (Milli-Q PF, Millipore, Schaffhausen, Switzerland) and sonicated for 15 min using a VCX 750 ultrasonicator (Sonics & Materials Inc., Newtown, CT, USA). Poly(methylmethacrylate) (PMMA) particles with a mesh size of about 32–50 μm were used as porogen and were prepared via free radical polymerization according to a procedure previously reported by Pradny et al. [30]. The chemical structures of materials used in this study are presented in Figure 1.

### 2.2. Sorbent Synthesis

The chemically cross-linked CS-based cryogels as monoliths (Figure 1D) were prepared at −20 °C by combining cryogelation with porogen leaching according to the method described previously by Dinu et al. (2013) [31]. Cryogelation was selected as a green and versatile approach for the synthesis of CS-based cryogels since the cross-linking reactions were performed in aqueous solutions. In addition, the cross-linking of CS with GA was performed in the presence of PMMA particles to generate porous architectures with less compact walls between macropores after leaching out the PMMA particles using acetone. The amount of GA varied between 2.5 *v*/*v*% and 7 *v/v*%, whereas the molar mass of CS (467 kDa), the concentration of CS solution (2 wt.%), the weight ratio between CS and PMMA particles, the mesh of PMMA particles, and the freezing/thawing conditions were kept constant during the preparation of all the cryogel samples. Typically, 0.64 mL of GA solution were first dropped over 10 mL of CS solution at a rate of 10 μL min^−1^ under vigorous stirring. This mixture was kept at 0 °C on an ice bath under continuous stirring for 30 min, and then PMMA particles (0.8 g) with a mesh size of about 32–50 μm were added into the CS/GA mixture when a paste was obtained. This paste was transferred into the chamber of a pelleting device, which was closed using a flange with fastening screws, and then incubated into a cryostat at −20 °C. After 24 h, the pelleting device was taken out from the cryostat and kept at room temperature for 1 h. The gels formed inside of the chamber of the pelleting device were carefully taken out and cut as monoliths that were 4 mm in length, which were then repeatedly immersed in a large excess of acetone to wash out the PMMA particles. All samples were washed with acetone for 7 days and finally with water–acetone mixtures whose content of water was increased as follows: 20% (2 h), 40% (2 h), 60% (2 h), 80% (2 h), and 100% (24 h). Thereafter, the swollen cryogels were frozen in liquid nitrogen and lyophilized for 24 h, at −57 °C and 0.045 mbar. The sample codes, the experimental parameters, and several other characteristics of the CS-based cryogels are summarized in Table 1.

### 2.3. Characterization of CSGA Cryogels

The gel fraction yield (*GFY*, %, Equation (S1)) [12], porosity (*P*, %, Equation (S2)) [32], and water uptake (*WU*, g g**^−1^**, Equation (S3)) [13] were calculated according to the equations presented in Table S1 in the Supplementary Materials. FTIR spectra were registered in the 4000–400 cm^−1^ range using a Vertex 70 FTIR spectrometer (Bruker, Ettlingen, Germany). All the samples were first mixed with KBr and then shaped as pellets by applying a force of 5 Nm. The ACD/Spec Viewer 5.04 Software was used to analyze the FTIR spectra. The internal morphology of the freeze-dried CSGA cryogels was evaluated using a Quanta 200-FEI environmental scanning electron microscope (FEI Company, Hillsboro, OR, USA) in low vacuum mode (20 kV). The Image J 1.48 v analyzing software was used to determine the average pore diameter of each sample [13]. The frequency of pores in each sample was expressed as a fraction in accordance with previous work on xanthan/polyvinyl alcohol cryogels [33]. The mechanical resistance to uniaxial compression was studied on swollen cryogel monoliths with a diameter of 8 mm and a height of 4 mm using an EZTest Shimadzu Testing Machine (Shimadzu, Kyoto, Japan). The setup and calculation of elastic moduli were done according to a previously published procedure [34]. All experiments were repeated three times and the average values ± SD are reported in the graphs. Contact angle (*θ*) measurements were performed to analyze the surface wettability of the CS-based cryogels using the drop technique, as indicated in [35]. Briefly, 1 μL of Milli-Q water was placed on the surface of each CS-based cryogel and investigated using a CAM-200 device (KSV Instruments Ltd., Helsinki, Finland). The *θ* values were determined by applying the Young–Laplace equation and the data are presented as the average of five consecutive measurements.

### 2.4. Batch Adsorption Experiments

Pesticide adsorption studies on CSGA cryogels were carried out using the batch technique. The CSGA cryogel samples were conditioned at pH 4.5 before the adsorption experiments to protonate the amine groups available for interaction with the Fastac 10EC pesticide. The contact time, sorbent dose, pH, and initial concentration of Fastac 10EC were varied to identify the appropriate adsorption conditions and achieve maximum pesticide removal. Thus, the effect of adsorption time was investigated in the 1–48 h time range, while the other parameters were kept constant. The effect of the sorbent dose on the Fastac 10EC removal was evaluated by changing the amount of cryogel from 0.004 to 0.034 g in the synthetic emulsions of Fastac 10EC. To study the influence of pH, the initial pH of the pesticide-containing wastewater was varied between 3 and 10 by adjusting its value with 0.1 M HCl or 0.1 M NaOH. Adsorption experiments were also performed using initial pesticide concentrations (*C_i_*, mg L^−1^) ranging from 0.09 mg L^−1^ to 0.36 mg L^−1^. To investigate the sorbent reusability, the pesticide loaded onto CSGA sorbents was eluted with 0.1 M HCl aqueous solution (0.05 L) for 1 h. Then, the CSGA sorbents were washed several times with distilled water and regenerated with 0.1 M NaOH aqueous solution (0.05 L) for 1 h. After this treatment, the sorbents were involved in another cycle of adsorption.

After each adsorption experiment, the CSGA cryogels were filtered off and the absorbance of residual pesticide in the filtrate was measured with a SPECOL 1300 Analytik Jena spectrophotometer at the wavelength of 276 nm (the maximum absorbance of Fastac emulsion). The pesticide removal efficiency (*RE*, %) was calculated with Equation (S4) (Table S1, Supplementary Materials) [36], while the amount of the Fastac 10EC pesticide adsorbed at equilibrium (*q_e_*, mg g^−1^) on all CSGA sorbents was determined using Equation (S5) (Table S1, Supplementary Materials) [12].

### 2.5. Antibacterial Study

The antibacterial activity of the CSGA cryogels was tested against one Gram-negative (*Escherichia coli*) and two Gram-positive bacterial strains (*Listeria monocytogenes* and *Staphylococcus aureus*), as reported previously [33,35,37]. All strains were ATCC-type Microbiologics KWIK-STIK and were purchased from Microbiologics. The antibacterial tests were performed in accordance with the International Standard ISO 11133.

## 3. Results and Discussion

### 3.1. Preparation and Characterization of CSGA Cryogels

CSGA cryogels with the chemical structure presented in Figure 1B were obtained by conducting the cross-linking reaction of CS in an apparently frozen system at −20 °C. To confirm the structures of the cryogels, FTIR spectroscopy was first used and the spectra of CS before and after the cross-linking were compared (Figures S1 and S2, Supplementary Materials). In the FTIR spectrum of the CS powder (Figure S1, Supplementary Materials), the characteristic absorption bands of CS were observed at 3460 cm^−1^, which was attributed to the stretching vibrations of –OH and –NH groups; 2963 cm^−1^ and 2874 cm^−1^, which corresponded to the asymmetric and symmetric vibration of –CH and –CH_2_ groups, respectively; and 1663 cm^−1^, which was assigned to the C=O bonds (amide I) [12,32]. In addition, the band at 1583 cm^−1^ was ascribed to the bending of –NH_2_ groups (amide II) [12,32]; the ones at 1420 cm^−1^ and 1383 cm^−1^ to the –CH_2_ bending and deformation, respectively; 1323 cm^−1^ was associated to the stretching vibration of C–N bonds (amide III) [12,32]; and the bands at 1155 cm^−1^, 1096 cm^−1^, and 1026 cm^−1^ were attributed to the C–O–C bridge deformations from the anhydroglucose (AGU) units and to the C–O stretching of secondary alcohol groups [12,32]. All these bands were also identified in the FTIR spectrum of the CSGA5 cryogel (Figure S2, Supplementary Materials) with some blue- or red-shifts. Thus, the bands at 3460 cm^−1^ and 2963 cm^−1^ in the FTIR spectrum of the CS powder were blue-shifted to 3441 cm^−1^ and 2922 cm^−1^, respectively, in the spectrum of the CSGA5 cryogel. The bands corresponding to amide I, amide II, and C-O stretching of secondary alcohol were blue-shifted to 1651 cm^−1^, 1564 cm^−1^, and 1078 cm^−1^, respectively, in the FTIR spectrum of CSGA5 cryogel, while the band corresponding to the C-O stretching vibrations of the secondary alcohol groups were red-shifted to 1038 cm^−1^ in the spectrum of CSGA5 cryogel. All these changes supported the evidence of successful cross-linking of CS with GA and the formation of chemically cross-linked CSGA cryogels.

**Figure 2:**
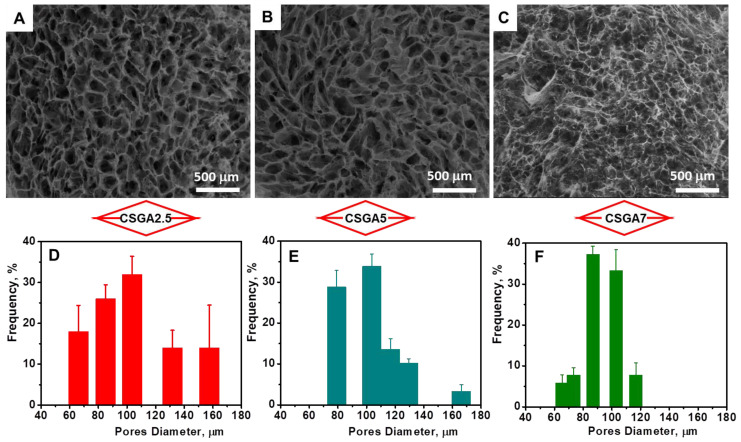
SEM micrographs and pore distribution diagrams of the CSGA2.5 (**A**,**D**), CSGA5 (**B**,**E**), and CSGA7 (**C**,**F**) cryogels.

The efficiency of cross-linking, i.e., *GFY* (Table 1), slightly increased with the increase in the amount of cross-linker from 78.40% for the CSGA2.5 cryogel to 79.43% for the CSGA7 cryogel. In addition, the *WU* values were also drastically influenced by the cross-linking degree. By increasing the cross-linking degree within the CS networks, the mobility and relaxation of the polymer chains were significantly hindered, which in turn impeded the accommodation of more solvent molecules within the CSGA matrices, and hence, lowered the equilibrium *WU*. Thus, at pH 2, the increase in cross-linker ratio decreased the *WU* values from 40.53 g g^−1^ for the CSGA2.5 cryogel to 20.18 g g^−1^ for the CSGA7 cryogel, while at pH 6, the *WU* values dropped off from 33.4 g g^−1^ for the CSGA2.5 cryogel to 15.78 g g^−1^ for the CSGA7 cryogel (Table 1). The porosities of the CSGA cryogels in a dried state (*P*, %) were evaluated using a solvent displacement technique and was calculated with Equation (S2) (Table S1, Supplementary Materials). The values of *P* were 95.17 ± 2.31%, 93.05 ± 1.67%, and 92.48 ± 0.78%, for the CSGA2.5, CSGA5, and CSGA7 cryogels, respectively, indicating the presence of a highly porous structure for all gels. The internal morphology of freeze-dried CSGA cryogels was further examined using SEM. Since the ice crystals acted as a template during the gel preparation, superporous architectures with interconnected pores were generated after gel thawing and drying (Figure 2A–C).

The use of PMMA particles as a porogen agent led to highly porous architectures with less compact walls (Figure 2A–C), whilst the CSGA cryogels prepared in the absence of PMMA exhibited a porous structure with more compact walls (Figure S3). The pore diameters and distribution within the 3D cryogel network (Figure 2D–F) were also influenced by the amount of cross-linker. As expected, the higher the cross-linker ratio, the smaller the pore size (Figure 2). Considering their prominent porosity and unique interconnected porous architecture, these sorbents allowed for faster diffusion of Fastac 10EC pesticide to the chelating functional groups (–NH_2_ and –OH) of the CS-based matrix.

**Figure 3:**
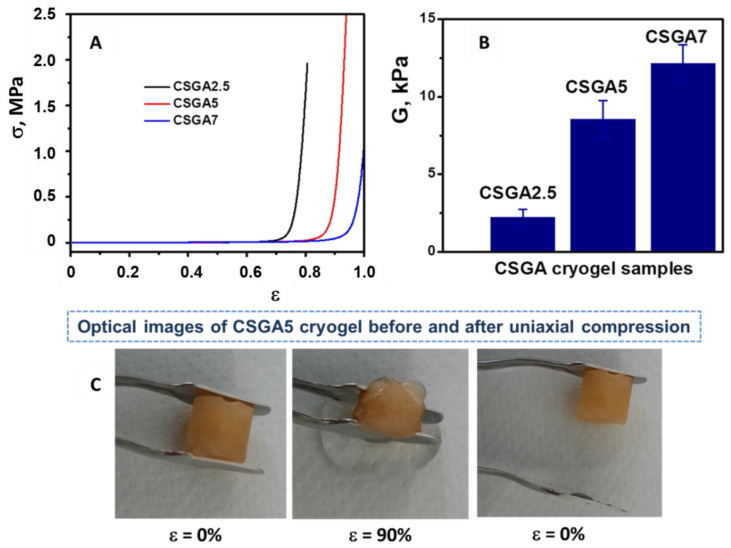
Stress–strain curves for the swollen CS-based cryogels (**A**); elastic moduli determined from the linear dependence of the stress-strain curves (**B**); optical images indicating the excellent elasticity and the complete shape recovery of a CSGA5 cryogel sorbent after compression (**C**).

The mechanical stabilities of CSGA cryogels were assessed using uniaxial compressive tests (Figure 3). The stress–strain profiles and the elastic modulus values of the CSGA cryogels in a swollen state are presented in Figure 3A,B.

All the CSGA cryogels were mechanically stable and sustained 80.64%, 94.23%, and 100% compression at compressive nominal stresses of 1.96 MPa, 2.75 MPa, and 1.83 MPa, respectively. As the CSGA cryogels were compressed between the parallel compression plates of the testing analyzer, the complete release of water from the pores of the gels hampered the crack development and gel fracturing at higher strain. In addition, the compressive modulus of all the CSGA cryogels increased with the increase in the cross-linker content (Figure 3B). Thus, the CSGA7 cryogel exhibited a five-times-higher elastic modulus than that of the CSGA2.5 cryogel, which was consistent with the formation of a much denser and stiffer cryogel network. Moreover, the pore size had a significant impact on the mechanical strength of the 3D porous matrix. As the presence of smaller pores results in an improvement in the compressive moduli [38,39], the enhancement in the elastic modulus for the CSGA7 cryogel (Figure 3B) may have been associated with the decrease in pore diameters (Figure 2). The optical images of the CSGA5 cryogel sorbent before and after 90% strain further supported the evidence of excellent elasticity and fast recovery of the original monolith shape after compression (Figure 3C). This behavior ensured an easier sorbent separation after adsorption; thus, the clean water was removed just by the simple compression of the cryogel sorbent, while the contaminant remained within the CS matrix.

### 3.2. Fastac 10EC Pesticide Adsorption

#### 3.2.1. Effect of Cross-Linking Degree, Contact Time, and Sorbent Dose

**Figure 4:**
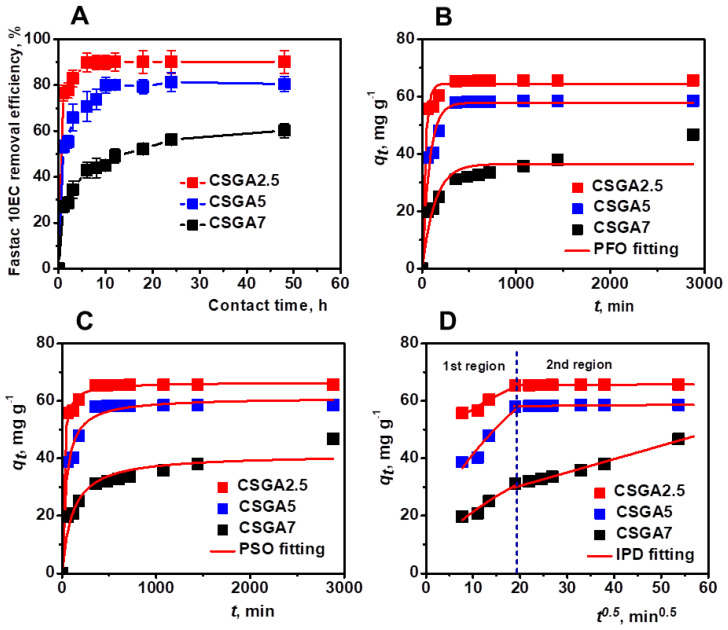
Effect of contact time and cross-linking degree on Fastac 10EC removal efficiency (**A**). Fastac 10EC adsorption experimental data fitted using PFO (**B**), PSO (**C**), and IPD (**D**) models. Adsorption conditions: pH 4.4, initial Fastac concentration of 0.18 mg L^−1^, sorbent dose 0.025 g.

The effect of contact time on Fastac 10EC removal efficiency using CSGA2.5, CSGA5, and CSGA7 cryogels was first monitored (Figure 4A). The adsorption of Fastac 10EC increased with time and then reached a plateau where no more pesticide was removed. The initial fast adsorption rate was associated with the abundance of vacant binding sites on the surface of CSGA sorbents. The cryogel with the lower cross-linking degree (CSGA2.5) exhibited the highest Fastac 10EC removal efficiency due to the presence of more –NH_2_ and –OH functional groups that were available for the interaction with pesticide molecules. This behavior agreed with the previous results reporting the removal of organochlorine pesticides by CS loaded with AgO nanoparticles [23] or the adsorption of ethoprophos on CS films [26]. Therefore, only the CSGA2.5 cryogel sorbent was further considered for Fastac 10EC removal.

To determine the Fastac 10EC adsorption rate and obtain more information on the process dynamics, the experimental kinetics data were fitted using pseudo-first-order (PFO, Equation (S6), Supplementary Materials, Figure 4B), pseudo-second-order (PSO, Equation (S7), Supplementary Materials, Figure 4C), and intraparticle diffusion (IPD, Equation (S8), Supplementary Materials, Figure 4D) models.

The experimental *q_t_* values for the Fastac 10EC removal using CSGA2.5, CSGA5, and CSGA7 sorbents were 65.68 mg g^−1^, 58.56 mg g^−1^, and 46.75 mg g^−1^, respectively (Figure 4B, Table 2).

The analysis of kinetics data indicated the PSO kinetic model as the most appropriate theoretical model due to its lower *χ*^2^ and higher *R*^2^ values (Table 2). The PSO kinetic model was also reported to describe the endosulfan adsorption onto poly(2-hydroxyethyl methacrylate-arginine methacrylate) cryogels [40] and the removal of atrazine pesticide by chitin-cl-poly(acrylamide-co-itaconic acid) hydrogels well [41].

The plot of *q_t_* vs. *t*^0.5^ pointed out the presence of two distinctive regions (Figure 4D). The first region showed the prevalent effect of IPD on Fastac 10EC adsorption. Since the number of active sites within the cryogel sorbents drastically decreased in the second region, the IPD effect became less significant, especially for the CSGA2.5 and CSGA5 sorbents. These results highlighted the interdependency between the Fastac 10EC adsorption rate and the availability of adsorption sites and indicated chemisorption as the removal mechanism.

**Figure 5:**
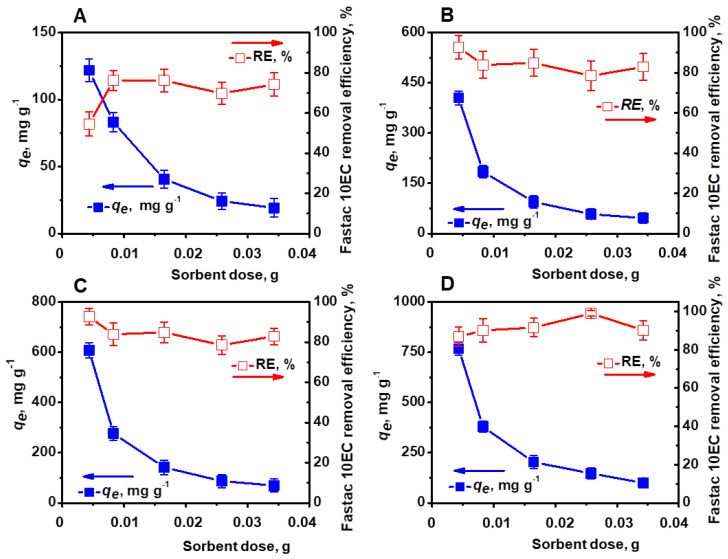
Effect of sorbent dose on the Fastac 10EC removal at various initial concentrations of pesticide: 0.09 mg L^−1^ (**A**), 0.18 mg L^−1^ (**B**), 0.27 mg L^−1^ (**C**), and 0.36 mg L^−1^ (**D**). Adsorption conditions: pH 6, contact time 24 h, temperature 22 ± 2 °C, and volume of 0.01 L.

The amount of sorbent is another important parameter that influences the affinity of a sorbent for a certain sorbate [23,26]. In this regard, the effect of sorbent concentration (0.004–0.034 g) on the removal of Fastac 10EC was investigated using the CSGA2.5 cryogel at various initial concentrations of pesticide (Figure 5).

The pesticide *RE* (%) was dependent on the initial pesticide concentration and the sorbent dosage. Thus, the maximum *RE* of 76.12% was obtained with about 0.01 g sorbent for a pesticide concentration of 0.09 mg L^−1^. A *RE*of 92.7% was achieved when 0.004 g sorbent was added to a pesticide concentration of 0.18 mg L^−1^ or 0.27 mg L^−1^, while for a concentration of 0.36 mg L^−1^, the maximum *RE*of 98.99% was acquired with 0.025 g sorbent.

#### 3.2.2. Effect of pH and Sorbent Reusability

**Figure 6:**
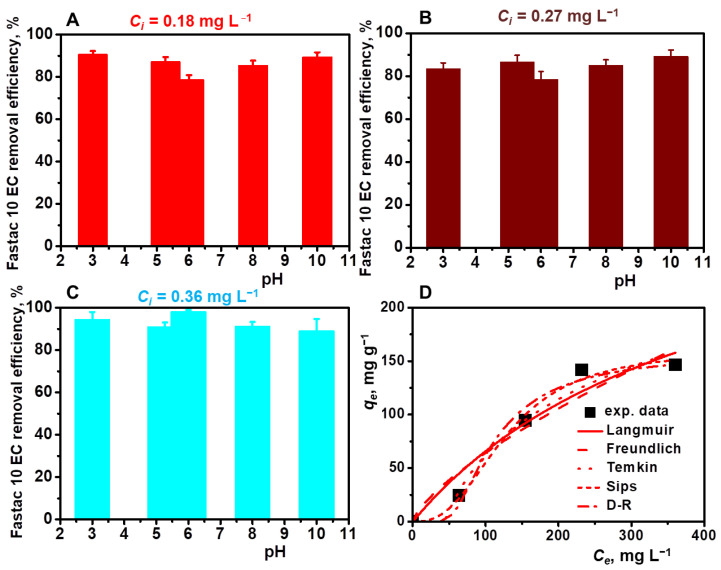
Effect of pH on the Fastac 10EC removal at various initial pesticide concentrations: 0.18 mg L^−1^ (**A**), 0.27 mg L^−1^ (**B**), and 0.36 mg L^−1^ (**C**). Equilibrium adsorption of Fastac 10EC onto CSGA2.5 cryogel sorbents fitted with Langmuir, Freundlich, Temkin, Sips, and D–R non-linear isotherm models (**D**). Adsorption conditions: sorbent dose of 0.025 g, contact time of 24 h, temperature of 22 ± 2 °C, and volume of 0.01 L.

The effect of pH on the Fastac 10EC adsorption process was monitored in the range of 3–10 at initial pesticide concentrations of 0.18 mg L^−1^, 0.27 mg L^−1^, and 0.36 mg L^−1^ (Figure 6A–C).

By increasing the initial pesticide concentration from 0.18 mg L^−1^ to 0.36 mg L^−1^, the Fastac 10EC removal efficiency varied between 80% and 97%, irrespective of the pH of the emulsion. Since CS-based cryogels were conditioned before the adsorption experiments at pH 4.5, below the *pK_b_* of CS, and the zeta potential values of pesticide formulation were between −24.6 mV in an acidic pH and -30 mV in a basic pH, the removal of Fastac 10EC was mainly driven by electrostatic interactions. This supported the successful application of CS-based cryogels for Fastac 10EC removal in both acidic and basic conditions. Similar behavior was previously indicated for CS–AgONPs composite sorbents, which completely removed the permethrin pesticide in the 3 to 11 pH range [23]. Our results are also in line with those reported for several dextran derivatives with quaternary ammonium groups in the main chain, where a *RE*of about 90% was obtained, irrespective of the pH value [42].

The experimental isotherms data were analyzed using Langmuir, Freundlich, Temkin, Sips, and Dubinin–Radushkevich (D–R) isotherm equations (Figure 6E and Table 3). The best fit was obtained using the Sips isotherm model, where a high correlation coefficient (0.992) and a maximum adsorption capacity of 160.82 mg g^−1^ were obtained. In addition, the value of energy calculated from the D-R isotherm was 17.85 KJ/mol (Table 3), which determined chemisorption as the mechanism for the adsorption of Fastac 10EC on the CSGA cryogel.

**Figure 7:**
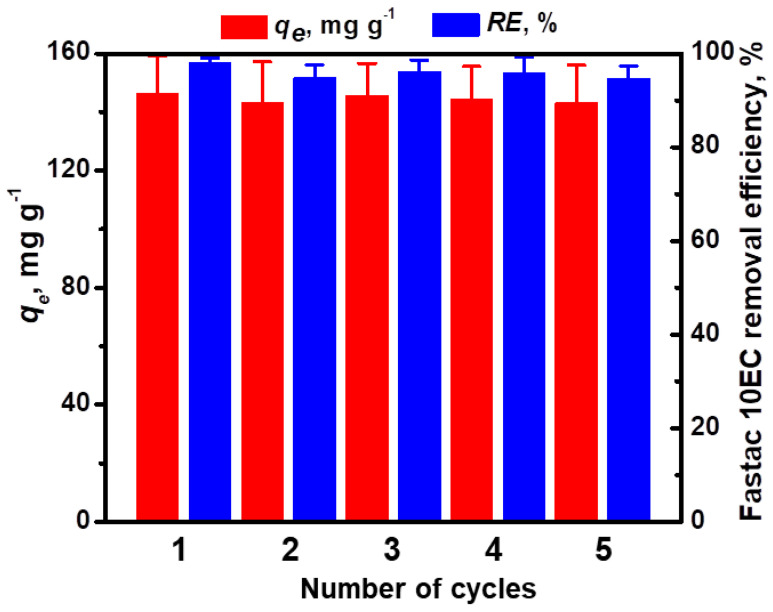
Reusability of CSGA2.5 cryogel sorbents for Fastac 10EC removal (pH 6, a sorbent dose of 0.025 g, and an initial Fastac 10EC concentration of 0.36 mg L^−1^).

The performance over multiple cycles of adsorption/desorption is an important feature that should be considered in the development of feasible, low-cost adsorbent materials [27]. To evaluate the reusability of our cryogel sorbents, we eluted the adsorbed Fastac 10EC pesticide with 0.1 M HCl aqueous solution for only 1 h and then regenerated the sorbent with 0.1 M NaOH aqueous solution. Since the elution of the pesticide was performed in the presence of a strong acid, the adsorption of Fastac 10EC into the CSGA matrix could be achieved again using chemisorption. Our results agree with those previously reported for the removal of methylene blue dye using alginate/clinoptilolite gel microbeads [13] and copper ions using CS-based ion-imprinted cryo-composites [27]. The reusability of CS-based cryogels for the removal of Fastac 10EC was investigated for five consecutive adsorption/desorption cycles (Figure 7).

The almost constant adsorption capacity, as well as the removal efficiency of Fastac 10EC at equilibrium after five cycles of adsorption/desorption, revealed the remarkable chemical stability of CS-based cryogels during the successive loading/leaching steps.

### 3.3. Antibacterial Activity

**Figure 8:**
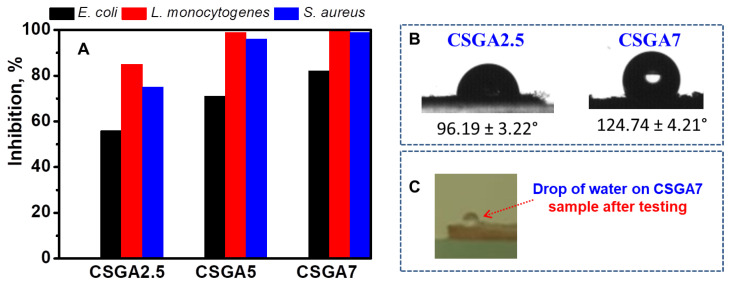
Antimicrobial activity of CS-based cryogels (**A**). Contact angle values for the CSGA2.5 and CSGA7 cryogels (**B**). Optical images of a water drop on the surface of a CSGA7 cryogel after the contact angle measurements (**C**).

Apart from pesticides, bio-contamination with various bacterial populations leads to waterborne diseases. Hence, it becomes vital to remove the above-mentioned contaminants from water using a suitable process. The disinfection studies using cryogels are currently scarce in the literature [43,44], and only one reported the antibacterial properties of hybrid cryogels based on CS, poly(dimethylamino ethylmethacrylate), and magnetite against *Escherichia coli* and *Streptococci*, besides the adsorption of arsenic and chromium from ground, surface, and drinking water [44]. To the best of our knowledge, we introduce here for the first time the ability of CS-based cryogels to inhibit the growth of one Gram-negative (*Escherichia coli*) and two Gram-positive bacteria, namely, *Listeria monocytogenes* and *Staphylococcus aureus* (Figure 8A), along with their remarkable adsorption capability for a pesticide. All CS-based cryogels were effective against the tested microorganisms, as indicated by the in vitro evaluation of the antibacterial activity. The bacteriostatic ability of a CS film is closely related to its physicochemical properties, such as charge density, water solubility, hydrophobicity, and chelating ability [45,46]. The main mechanism of CS action is based on the electrostatic interaction between the positive charges of amino groups present in CS and the negative charges on the microbial surface [45,46]. In this study, CS-based cryogels exhibited better antimicrobial activity against Gram-positive than Gram-negative bacteria due to the non-covalent interaction of CS with teichoic acid from the peptidoglycan layer, which finally led to the disruption of cell functions and subsequent cell death [47].

However, the CSGA7 cryogel showed a more promising antimicrobial activity against all tested microorganisms when compared with the CSGA2.5 cryogel. Since the CSGA7 cryogel exhibited a low water swelling and high contact angle values (Table 1 and Figure 8B,C), it was apparent that its pronounced hydrophobicity had a major contribution to the enhanced inhibitory effect. Similar behavior was previously reported for xanthan/polyvinyl alcohol/Merlot pomace-based cryogels [37].

## 4. Conclusions

In this study, we successfully investigated the adsorption performance of highly porous, chemically cross-linked 3D chitosan cryogels (CSGA) toward the Fastac 10EC pesticide. All the CSGA cryogels were mechanically stable and sustained 80.64%, 94.23%, and 100% compression at compressive nominal stresses of 1.96 MPa, 2.75 MPa, and 1.83 MPa, respectively. This remarkable elasticity guaranteed the facile separation of the sorbent after the adsorption process; thus, the clean water was leached out of the cryogel sorbent just via simple compression, while the contaminant remained within the CS matrix. The Fastac 10EC pesticide was removed using CSGA cryogels with high efficiency (80–97%) in both acidic and basic conditions. The equilibrium data were well fitted using the Sips model with a theoretical maximum adsorption capacity of 160.82 mg Fastac 10EC/g sorbent. In addition, CS-based cryogel sorbents showed remarkable stability during successive adsorption/desorption cycles. The kinetics, adsorption isotherms, and reusability data identified chemisorption as the mechanism responsible for the Fastac 10EC removal. Furthermore, the CS-based cryogels exhibited an outstanding activity in inhibiting pathogenic bacteria, namely, *Escherichia coli*, *Listeria monocytogenes*, and *Staphylococcus aureus*. Our means of preparing CS-based cryogels as sorbents with antimicrobial properties paves the way for technological approaches that might be extremely useful in environmental applications since they are: (i) prepared using an easy and eco-friendly strategy (cross-linking reactions conducted in aqueous solutions), (ii) efficient in both acidic and basic media, (iii) stable under several successive adsorption/desorption cycles without loss of activity, (iv) rapidly separated after adsorption just via simple compression, (v) not soluble in water (essential for water-disinfection applications), and (vi) effective in suppressing the growth of a broad spectrum of pathogenic microorganisms. Since real wastewaters consist of complex mixtures of inorganic and organic pollutants, further studies are necessary to evaluate the removal of pesticides from multicomponent mixtures with heavy metal ions, clays, silicates, or metal oxides. Furthermore, the preparation of these cryogels at a large scale will have to be taken into consideration in order to handle and treat larger volumes of pesticide-containing wastewaters resulting from industrial and agriculture activities.

## Figures and Tables

**Table 1:** The sample codes, the feed composition, and some characteristics of the CS-based cryogels.

Sample Code	Feed Composition			*GFY*, ^b^ %	*P*, ^c^ %	*WU*, ^d^ g g^−1^	
	CS, wt.%	GA, *v*/*v*%	CS: PMMA, ^a^			pH 2	pH 6
CSGA2.5	2	2.5	1:4	78.40 ± 1.61	95.17 ± 2.31	40.53 ± 3.42	33.40 ± 0.84
CSGA5	2	5	1:4	79.10 ± 2.07	93.05 ± 1.67	28.07 ± 1.19	20.51 ± 1.92
CSGA7	2	7	1:4	79.43 ± 0.82	92.48 ± 0.78	20.18 ± 0.23	15.78 ± 1.46

^a^ The mesh size of PMMA particles was approximately 32–50 μm; ^b^ *GFY*—gel fraction yield; ^c^ *P*—porosity; ^d^ *WU*—water uptake.

**Table 2:** Kinetic model parameters for the adsorption of Fastac 10EC onto CSGA sorbents.

Kinetic Models	Parameters	CSGA2.5	CSGA5	CSGA7
	*q_t,exp_*, mg g^−1^	65.68 ± 3.62	58.56 ± 2.36	46.75 ± 2.41
PFO	*q_t,calc_*, mg g^−1^	64.47 ± 0.93	57.85 ± 1.29	36.50 ± 1.97
	*k*_1_, min^−1^	0.029 ± 0.004	0.013 ± 0.006	0.007 ± 0.002
	*R* ^2^	0.983	0.967	0.871
	*χ* ^2^	7.144	11.564	21.52
PSO	*q_t,calc_*, mg g^−1^	66.48 ± 0.62	61.21 ± 1.24	41.37 ± 2.06
	*k*_2_, g mg^−1^ min^−1^	10.8 × 10^−4^	3.8 × 10^−4^	2.2 × 10^−4^
	*R* ^2^	0.995	0.983	0.940
	*χ* ^2^	1.93	6.03	10.14
IPD	*k_id._*_1_, mg g^−1^min^0.5^	6.47 ± 1.20	0.56 ± 8.59	7.64 ± 1.33
	*C*_1_, mg g^−1^	36.68 ± 4.29	12.94 ± 2.40	−2.74 ± 4.75
	*R* ^2^	0.935	0.935	0.943
	*χ* ^2^	1.88	7.52	2.31
	*k_id._*_2_, mg g^−1^min^0.5^	0.007 ± 0.002	0.013 ± 0.004	0.467 ± 0.026
	*C*_2_, mg g^−1^	65.37 ± 0.07	57.94 ± 0.134	21.08 ± 0.92
	*R* ^2^	0.860	0.860	0.994
	*χ* ^2^	0.051	0.101	0.693

**Table 3:** Isotherm parameters and coefficients of determination for the adsorption of Fastac 10EC by a CSGA2.5 cryogel sorbent.

Isotherm	Parameters	CSGA2.5 Cryogel Sorbent
Langmuir	*q_m_*, mg g^−1^	340.90
	*K_L_*, L mg^−1^	0.0024
	R_L_	0.864
	*R* ^2^	0.946
	*χ* ^2^	321.35
Freundlich	*K_F_*, mg g^−1^	2.32
	1/*n*	0.72
	*R* ^2^	0.925
	*χ* ^2^	445.24
Temkin	*b_T_*, J mol^−1^	32.45
	*a_T_*, L mg^−1^	0.023
	*R* ^2^	0.976
	*χ* ^2^	141.86
Sips	*q_m_*, mg g^−1^	160.82
	*a_S_*	2.73 × 10^−6^
	1/*n*	2.64
	*R* ^2^	0.992
	*χ* ^2^	65.86
Dubinin–Radushkevich (D–R)	*q_DR_*, mg g^−1^	157.75
	*b*, mol^2^ kJ^−2^	0.0016
	*E*, kJ mol^−1^	17.85
	*R* ^2^	0.983
	*χ* ^2^	101.88

## Data Availability

Not applicable.

## Supplementary Materials

The following supporting information can be downloaded from https://www.mdpi.com/article/10.3390/polym14153145/s1; the supporting information contains FTIR, SEM micrographs, and experimental adsorption data analysis regarding the kinetics and isotherms. Table S1: Equations used for evaluation of CSGA cryogel features; Figure S1: FTIR spectrum of CS powder; Figure S2: FTIR spectrum of CSGA5 cryogel; Figure S3: SEM micrographs of CSGA cryogels at magnifications of 25× (A) and 100× (B), prepared in the absence of PMMA particles.
