# Supplementary material for: Macroporous 3D Chitosan Cryogels for Fastac 10EC Pesticide Adsorption and Antibacterial Applications
Source: Polymers (Basel). 2022 Aug 2;14(15):3145. doi: 10.3390/polym14153145 (PMC9370839; doi:10.3390/polym14153145)
Supplement: Supplementary file 1 [file polymers-14-03145-s001.zip › polymers-1825859-supplementary.pdf]

Supporting information

Macroporous 3D chitosan cryogels for Fastac 10 EC pesticide adsorption and antibacterial applications

Ionel Adrian Dinu,[1]* Luminita Ghimici,[1] Irina Elena Raschip[1]

[1]"Petru Poni" Institute of Macromolecular Chemistry, Grigore Ghica Voda Alley 41A, Iasi 700487, Romania

*Corresponding author: Dr. Ionel Adrian Dinu, *E-mail address*: adinu@icmpp.ro

Table S1. Equations used for evaluation of CSGA cryogel features.

Eq. no.	Equation	Description	Refs.[a]
(S1)	$GFY(\%) = \frac{W_d}{W_m} \times 100$	W_d –amount of cryogel after freeze-drying, g; W_m –total amount of compounds used for preparation of cryogels, g.	[12]
(S2)	$P\ (\%) = \frac{V1 - V3}{V2 - V3} x\ 100$	V_1 –initial volume of isopropanol, L; V_2 – total volume of isopropanol containing the immersed cryogels, L; V_3 – volume of isopropanol after the cryogel removal, L.	[31]
(S3)	$WU\ (g/g)\ = \frac{Wt\ -\ Wd}{Wd}$	W_t – weight of cryogel in swollen state at time *t*, g; W_d – weight of cryogel in dried state, g.	[13]
(S4)	$RE(\%) = \frac{Ci - Cf}{Ci}\ X\ 100$	*RE* – removal efficiency, %; C_i – initial pesticide concentration, mg L^{-1}; C_f – pesticide concentration after the interaction with cryogels, mg L^{-1}.	[35]
(S5)	$q_e = \frac{(C_i - C_e) \times V}{m}$	q_e – amount of Fastac 10EC adsorbed at equilibrium, mg g^{-1}; C_i – the same meaning as in Eq. (4); C_e – pesticide concentration at equilibrium, mg L^{-1}; *V* – volume of aqueous emulsion, L; *m* – sorbent dose, g.	[12]

[a]References numbers from the main text.

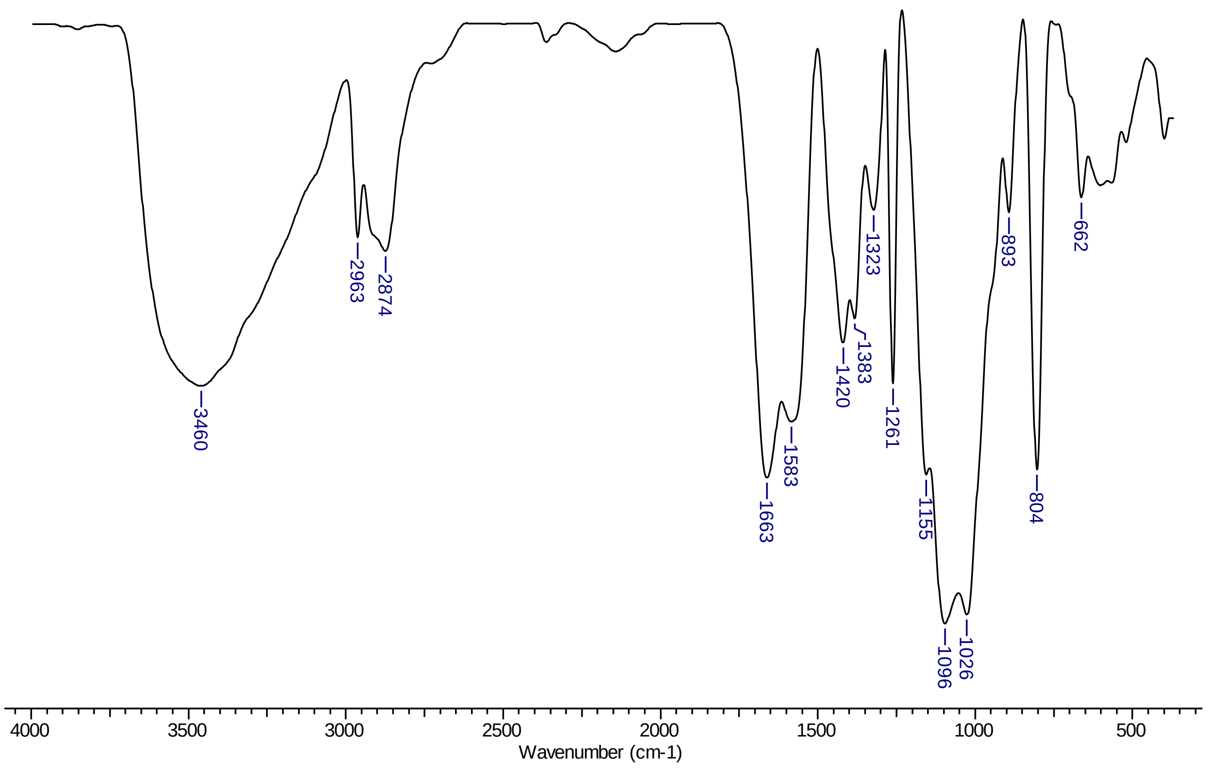

Figure S1. FTIR spectrum of CS powder.

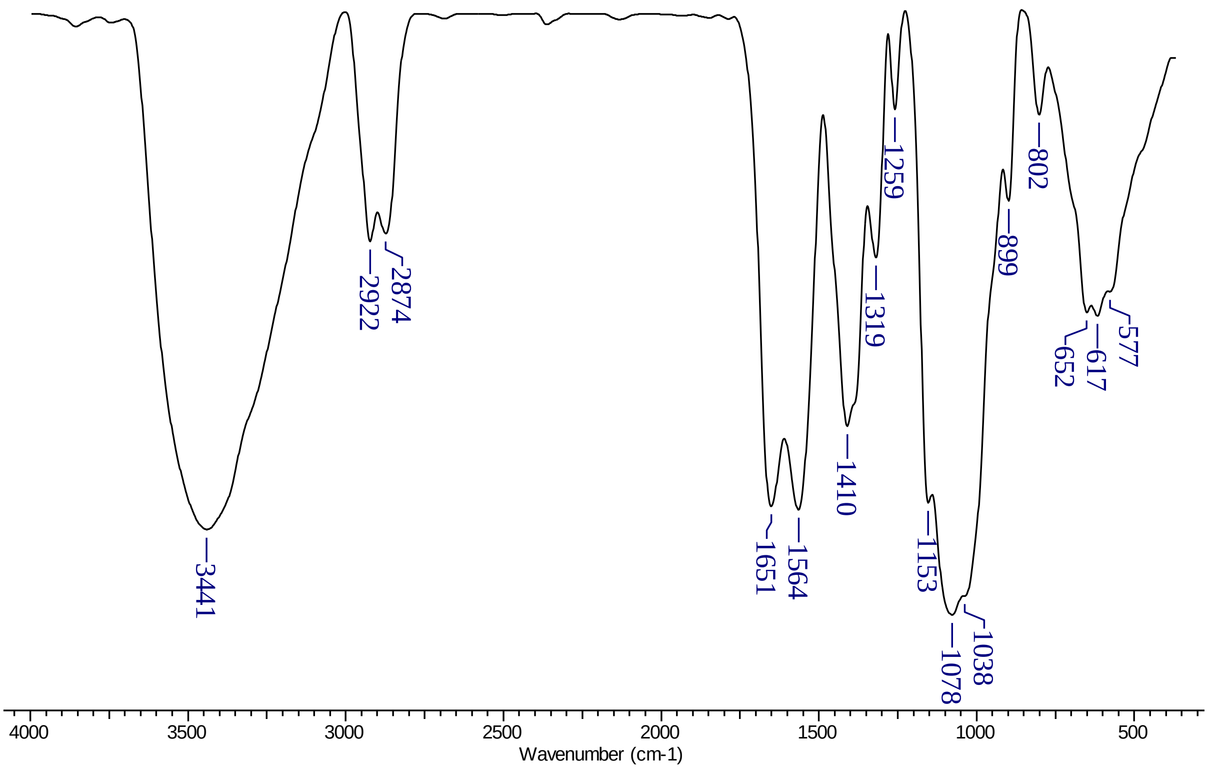

Figure S2. FTIR spectrum of CSGA5 cryogel.

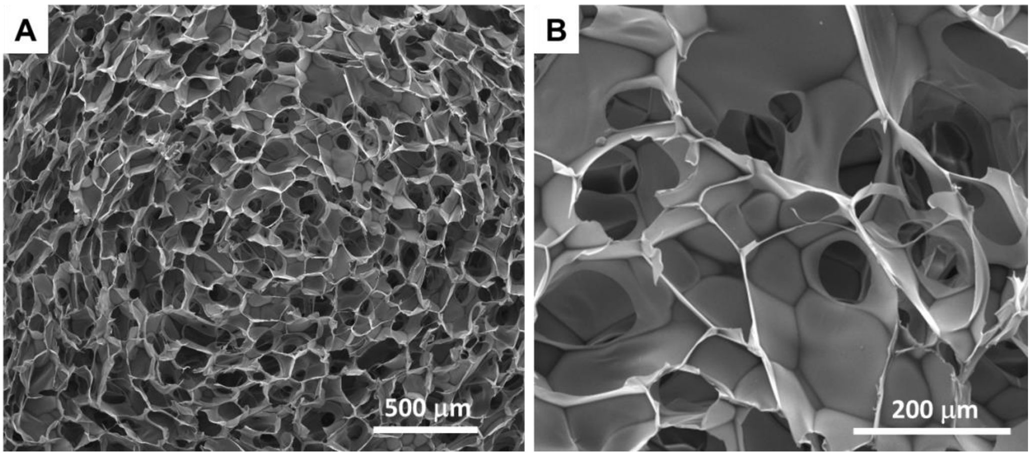

Figure S3. SEM micrographs of CSGA cryogels at magnifications of 25x (A) and 100x (B), prepared in the absence of PMMA particles.

Experimental sorption – data analysis

1. Kinetic equations

1.1. Pseudo-first order (PFO) model:

$$q_t = q_e(1 - exp^{-k_1 t}) \quad \text{(S6)}$$

where q_e and q_t – amount of pesticide adsorbed at equilibrium and at time t, min, respectively; k_1 – rate constant of the PFO kinetic model, min^{-1}.

1.2. Pseudo-second order (PSO) model:

$$q_t = \frac{q_e^2 k_2 t}{1 + q_e k_2 t} \quad \text{(S7)}$$

where q_e and q_t have the same meaning as in Eq. 6; k_2 – rate constant of the PSO kinetic model, g mg^{-1} min^{-1}.

1.3. Intraparticle diffusion (IPD) model:

$$q_t = k_{id} t^{0.5} + C \quad \text{(S8)}$$

where q_t has the same meaning as in Eq. 6; k_{id} – intraparticle diffusion rate constant, mg g^{-1} min$^{-1/2}$; C – constant describing the effect of boundary layer thickness, mg g^{-1}

2. Isotherm equations

2.1. Langmuir model:

$$q_e = \frac{q_m K_L C_e}{1 + K_L C_e} \quad \text{(S9)}$$

where q_e has the same meaning as in Eq. 6; q_m – maximum theoretical sorption capacity, mg g^{-1}; K_L – Langmuir constant, L mg^{-1}; C_e – concentration of the Fastac 10EC in aqueous emulsion at equilibrium, mg L^{-1}.

2.2. Freundlich model:

$$q_e = K_F C_e^N \quad \text{(S10)}$$

where q_e has the same meaning as in Eq. 6; K_F – Freundlich constant, mg g^{-1} mg^{-N} L^{-N}; C_e has the same meaning as in Eq. 9; N – measure of both the nature and strength of adsorption process, and of active

sites distribution, related to the surface heterogeneity; the larger is its value, the more heterogeneous the sorbent system is.

2.3. Sips model:

$$q_e = \frac{q_m a_S C_e^N}{1+a_S C_e^N} \tag{S11}$$

where q_e, q_m, C_e have the same meaning as in Eqs. 6 and 9; a_S – Sips constant.

2.4. Temkin model:

$$q_e = \frac{RT}{b_T}\ln(a_T C_e) \ and \ E_T = \frac{RT}{b_T} \tag{S12}$$

where q_e, C_e have the same meaning as in Eqs. 6 and 9; b_T is the Temkin constant, a_T (L g^{-1}) is the equilibrium binding constant, while E_T (kJ mol^{-1}) is related to the heat of adsorption; R – gas constant, J mol^{-1} K^{-1}; T – temperature, K.

2.5. Dubinin–Radushkevich (D–R) model:

$$q_e = q_{DR} e^{-K_{DR}\varepsilon^2} \tag{S13}$$

where q_e has the same meaning as in Eq. 6; q_{DR} – maximum sorption capacity of the pesticide, mg g^{-1}; K_{DR} – D–R isotherm constant, mol^2 kJ^{-2}; Polanyi potential:

$$\varepsilon = RTln\left(1+\frac{1}{C_e}\right) \tag{S14}$$

C_e, R, T have the same meaning as in Eqs. 9 and 12, respectively.
